# Lethal Complication From Inappropriately Prepared Polyethylene Glycol (Golytely) in a Pediatric Patient

**DOI:** 10.7759/cureus.13713

**Published:** 2021-03-05

**Authors:** Chahdah Skaff, Tareq Alayed

**Affiliations:** 1 Pediatric Critical Care Medicine, Dr. Sulaiman AL Habib Medical Group, Riyadh, SAU; 2 Pediatric Critical Care Medicine, King Faisal Specialist Hospital and Research Center, Riyadh, SAU

**Keywords:** pediatric, polyethylene glycol, esophageal atresia repair, colonic interposition

## Abstract

Polyethylene glycol with electrolytes (PEG-ELS, Golytely) is a widely used osmotic solution for colonic preparation in adults who are undergoing colonoscopy or colon surgeries. In pediatric patients, It is approved for the same indications and as a treatment for severe chronic constipation. PEG-ELS has an acceptable safety profile and minimal side effects. The most common adverse effects are nausea and abdominal cramps. Golytely is known to cause minimal electrolyte disturbances with no major sequelae. A few case reports have been published describing the effect of PEG-ELS on the electrolytes.

In this report, we present a case of an inappropriate preparation of Golytely administered to a pediatric patient leading to severe electrolyte disturbances and death.

## Introduction

Polyethylene glycol with electrolytes (PEG-ELS, Golytely) is an osmotically balanced, high-volume, non-absorbable electrolytes solution. It is commonly used in pediatric patients as a treatment for chronic severe constipation [1] and in the preparation for colonoscopy [2]. Its safety profile encourages pediatricians to use it widely. The main reported side effects are nausea, vomiting, and abdominal pain [3]. Golytely causes minimal electrolytes shifting when prepared and administered appropriately.

In this report, we discuss the case of a child who was known to have had a complicated post-esophageal atresia repair. He had undergone many corrective surgeries but ended up with severe esophageal strictures. He was scheduled for colonic interposition surgery. One of the main preoperative preparations involved colonic cleansing using an osmotic agent. Inadvertently, the child received an inappropriately made Golytely preparation. This led to severe diarrhea and serious hypernatremic dehydration. Unfortunately, the child's condition deteriorated rapidly, leading to multiorgan dysfunction and brain death.

## Case presentation

Our patient was a five-year-old boy who was known to have esophageal atresia and tracheoesophageal fistula post multiple surgical repairs. His course of therapy had been complicated by recurrent tracheoesophageal fistulae, which had required different modalities of surgical therapy. He ended up with a proximal esophagectomy and a closed distal esophagus.

A year later, he was scheduled to undergo colonic interposition for esophageal replacement. He was admitted to a non-surgical adult unit due to the unavailability of pediatric beds. The plan was to start bowel preparation overnight using Golytely and bisacodyl. The approved protocol was to administer 25 ml/kg/hour of diluted Golytely the night before the surgery. This dose could be repeated until the output was clear. During this time, the child would be fasting and intravenous fluids (IVF) would be started. Inadvertently, at 22:00 on 9/12, an undiluted 375 ml of Golytely was administered over one hour through a gastrostomy. IVF was started four hours after starting the laxative. Six hours after the administration, at 04:00 on 10/12, the child passed a large amount of watery stool. An hour later, he became lethargic, had poor peripheral perfusion, and became tachycardic.

His vital signs before starting Golytely and at the time of the event are presented in Table 1.

**Table 1:** Vital signs before and during the event

Vitals	Before Golytely (9/12, 20:00)	At the time of the event (10/12, 04:00)
Temperature (degree celsius)	36.6	37.5
Heart rate (beats per minute)	91	210
Respiratory rate (breaths per minute)	22	52
Saturation (%)	98	99
Blood pressure, mmHg	105/61	90/70

His condition deteriorated rapidly and his level of consciousness dropped, requiring the caregivers to secure his airway. His heart rate was persistently high and he developed high-grade fever (39.9 °C). His ECG showed evidence of supraventricular tachycardia, for which he received three doses of adenosine and two synchronized cardioversion. During the resuscitation, he received a total of 100 ml/kg of crystalloids and colloids. He was then shifted to the pediatric intensive care unit (PICU) with the following vitals: temperature: 37.5 °C, heart rate 159 beats per minute, and blood pressure: 91/54 mmHg.

The trend of his blood gas levels is shown in Table 2.

**Table 2:** Blood gas parameters ABG: arterial blood gas; VBG: venous blood gas; HCO_3_: bicarbonate

Parameters	ABG (10/12, 04:15)	VBG (two hours later: 06:15)	VBG (six hours later: 13:00)
pH	7.4 (7.35-7.45)	7.14 (7.33-7.43)	7.21 (7.33-7.43)
pCO_2_ (kPa)	3.7 (4-5)	8.6 (6-6.8)	4.2 (6-6.8)
pO_2_ (kPa)	12.7 (10-13)	5.4 (4.7-5.3)	8.2 (4.7-5.3)
HCO_3_ (mmol/L)	17.3 (22-26)	22.1 (24-28)	12.4 (24-28)
Lactate (mmol/L)	3.9 (0.5-1)	4.9 (0.5-1)	5.7 (0.5-1)

The labs before and after the event are presented in Table 3.

**Table 3:** Laboratory values before and after the event CBC: complete blood count; WBC: white blood cells; Hb: hemoglobin; HCT: hematocrit; ALT: alanine aminotransferase; AST: aspartate aminotransferase; ALP: alkaline phosphatase

Labs	Before the event (9/12, 11:30)	After the event (10/12, 04:20)
CBC		
WBC (10^3^/mL) (5-15)	4.85	22.4
Hb (mg/dL) (110-140)	143	185
HCT (33-42%)	0.423	0.547
Platelets (10^3^/mL) (150-400)	265	504
Renal function		
Urea (mmol/L) (2.5-8)	5	7
Creatinine (mmol/L) (28-52)	22	35
Sodium (mmol/L) (136-145)	137	171
Chloride (mmol/L) (98-107)	101	131
Potassium (mmol/L) (3.5-5)	4.7	4.1
Bicarbonate (mmol/L) (20-28)	18	15
Phosphate (mmol/L) (1.05-1.85)	--	0.75
Liver function		
ALT (IU/L) (<55)	15.1	22.4
AST (IU/L) (5-34)	30	51.1
ALP (IU/L) (142-335)	144	186
Albumin (g/L) (38-54)	41.6	48

**Figure 1:**
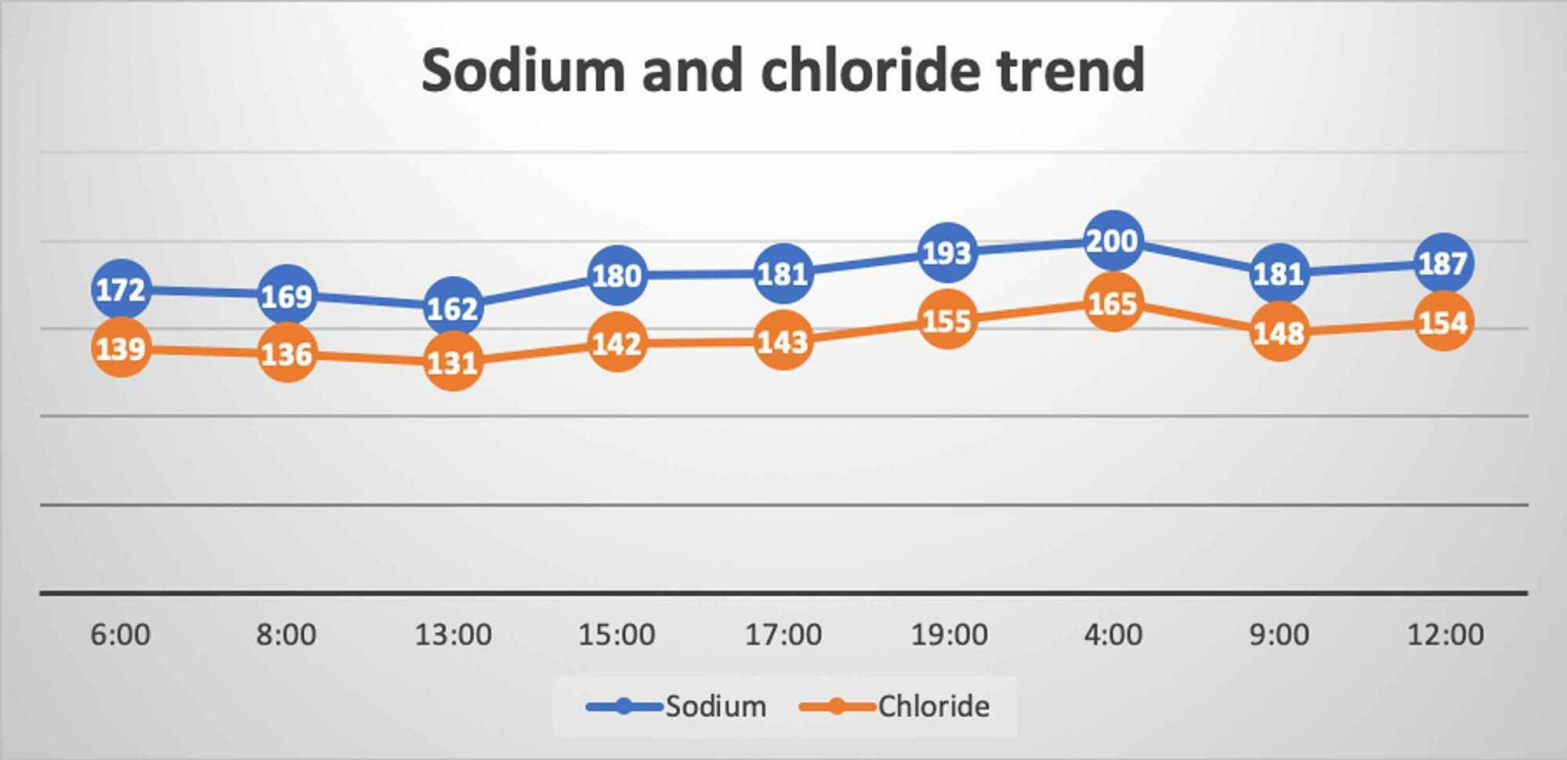
Trend of sodium and chloride This figure demonstrates the progressive elevation in sodium and chloride over 36 hours

**Figure 2:**
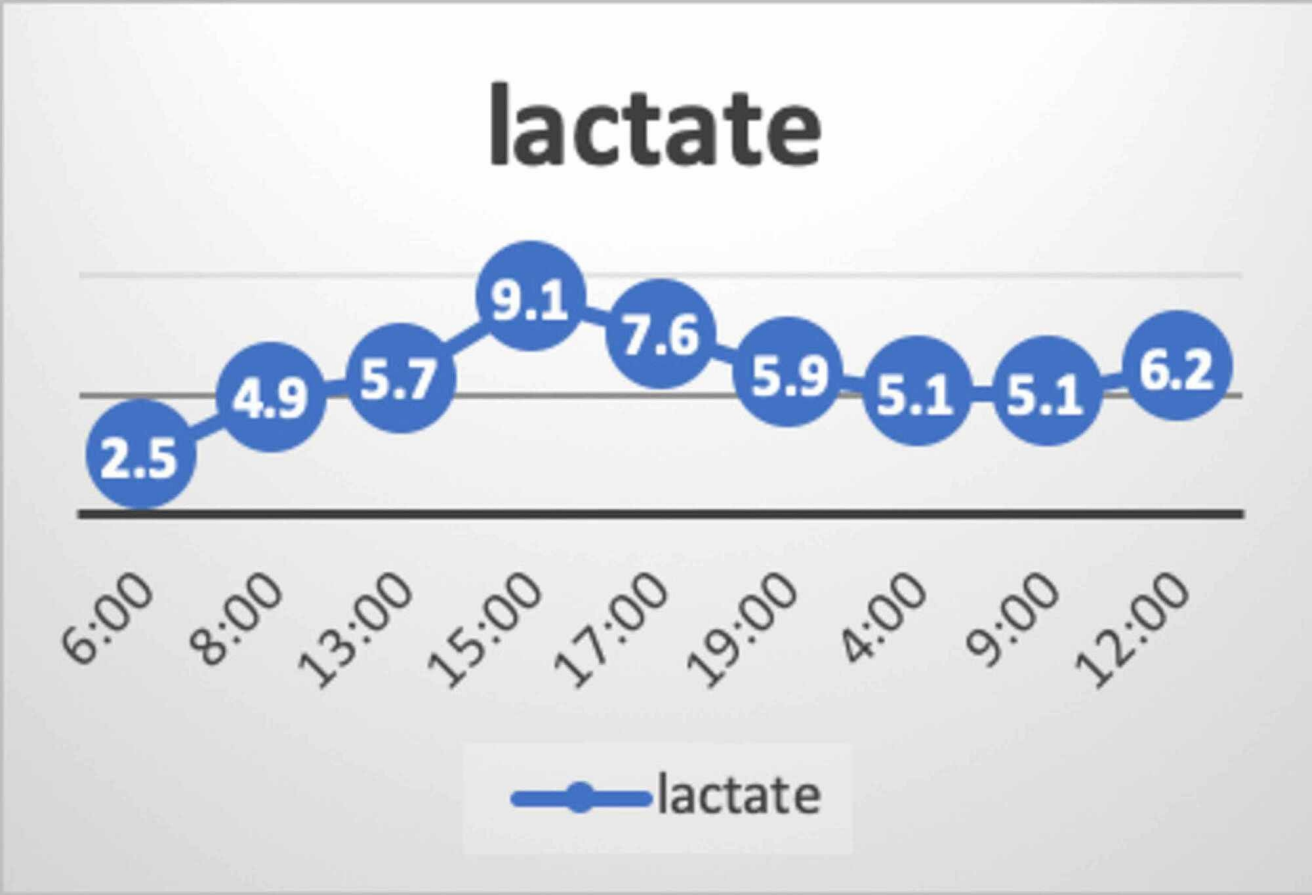
Trend of lactate This figure shows the lactate levels over 36 hours. It shows a progressive elevation of lactate within the first 12 hours, followed by gradual improvement, and then re-elevation

**Figure 3:**
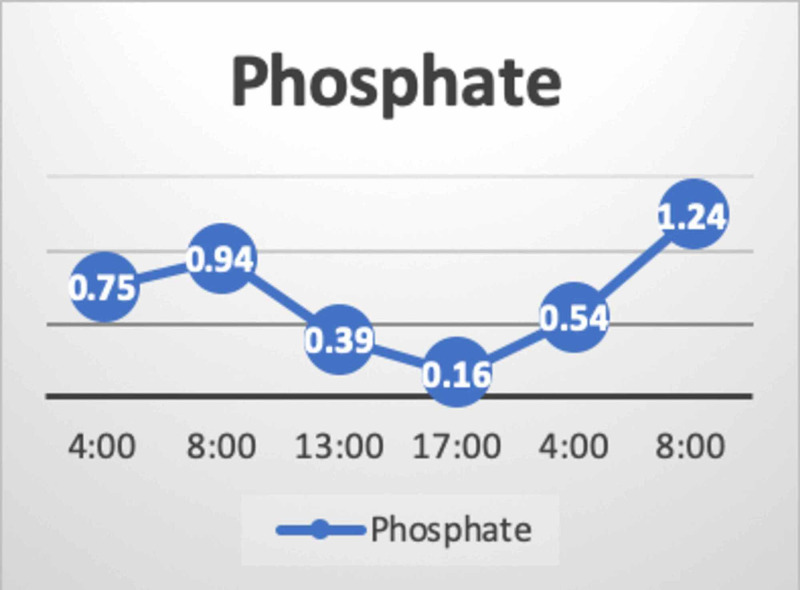
Trend of phosphate The figure represents the trend of phosphate over a period of 26 hours. The phosphate level reached its nadir within 12 hours from the event; it was difficult to correct initially and then started to improve with replacement

The patient's initial labs showed significant hemoconcentration and severe hypernatremia. His plasma osmolality was 409 mOsm/Kg (normal range: 275-290 mOsm/Kg) and his urine osmolality was 220 (normal range: 50-1200 mOsm/Kg), 12 hours after the event. The inflammatory markers were benign: procalcitonin of 0.23 and CRP of 5, and no positive cultures were reported. The child was empirically started on broad-spectrum antibiotics to cover the possibility of sepsis. His condition continued to deteriorate and he developed multiorgan dysfunction syndrome (MODS). The striking features of his labs at that time were the progressive increment in sodium, chloride, lactate, and the decline in phosphate despite optimizing his intravascular volume. The trends of sodium, chloride, lactate, and phosphate over 24 hours are presented below (Figure 1, Figure 2, Figure 3).

Six hours later, the child developed pulseless electrical activity (PEA); effective cardiopulmonary resuscitation (CPR) was performed, and restoration of spontaneous circulation (ROSC) was achieved after nine minutes. Afterward, he required high inotropic supports. Four hours later, he developed bilateral dilated, non-reactive pupils. An urgent CT scan was done and showed severe brain edema with tonsillar herniation. Neuroprotective measures were applied. But unfortunately, his condition kept deteriorating, and the child developed ventricular tachycardia and passed away.

## Discussion

Appropriate bowel preparation is an important step in any major colon surgery, such as colonic interposition in complicated esophageal atresia in pediatric patients. The ideal substance for colonic cleanout should be safe and effective, and should not cause any changes in the colonic structure or any electrolytes derangements [4].

In 1980, Davis et al. developed PEG-ELS-based laxatives (Golytely), which later became the most commonly used solution for colonic cleanout in both adults and pediatric patients [5]. PEG-ELS is a balanced preparation that travels through the gastrointestinal tract without absorption or secretion of electrolytes or fluids. Hence, colonic cleansing happens due to the mechanical drag of stool by fluid load [5]. In 1988, Schiller et al. studied the osmotic effect of PEG and concluded that it exhibits a greater osmotic effect than can be accounted for by the number of PEG molecules found in the preparation [6].

Hammer et al. compared osmotic diarrhea induced by PEG vs. lactulose. They concluded that the higher osmotic loads of PEG resulted in a near-linear increment in stool weight and water output. They also found that PEG-induced diarrhea was associated with minimal enteral loss of sodium, potassium, and chloride [7]. Since PEG does not cause major electrolytes and fluid derangement, it gradually became the preferred agent for colonic preparation in pediatric patients. In a survey conducted by the NASPGHAN group in 2014, 80% of responders reported using PEG with or without a stimulant for bowel cleanout. The most common stimulants used were bisacodyl and Senna. In the same survey, it was observed that there were wide differences in the dosage of PEG prescribed by different physicians [8]. The usual protocol in pediatrics is to give 25 ml/kg of Golytely per hour, with a maximum dose of 450 ml per hour, until the stool is clear. It usually takes four hours to complete the preparation [8,9]. Golytely is available as a powder component in a 4-L container. This content should be diluted in 4 L of water. Afterward, the prescribed amount will be given to the child. The 4-liter diluted Golytely contains 59 g/L of PEG, 5.69 g/L of sodium sulfate, 1.69 g/L of sodium bicarbonate, 1.47 g/L of sodium chloride, and 0.743 g/L of potassium chloride [10].

Over the years, Golytely has gained widespread acceptance among pediatricians due to its better palatability compared to other products. However, a good number of pediatric patients require the insertion of a nasogastric tube (NGT) to consume the required amount over a specific period of time, which is usually difficult in very small pediatric patients [9]. In a review, Peña and Bischoff have described the usual colorectal preparation using Golytely for colon surgeries. Cleaning the colon takes approximately four hours. During this time, the patient is allowed to take clear fluids only. In small children of less than two years, it is recommended to start IVF during the process. However, in children aged more than two years, it is desirable to have IVF, but not mandatory. The reason is to prevent the development of dehydration and metabolic acidosis that occur in pediatric patients during the process of colonic preparation [9]. Many studies have confirmed the safety of PEG use in pediatric patients. The most common reported side effects are abdominal discomfort, bloating, nausea, and vomiting [3]. Even in a reported case of unintentional IV administration of PEG, there were no significant side effects after aggressive fluid management [11].

A review of the literature revealed that there are a few studies reporting more severe and sometimes lethal side effects of PEG. In a case series published in 2017, 12 cases of adverse effects of PEG were reported. Seven of these cases were pediatric patients. All of them developed aspiration pneumonitis post administration of PEG through NGT. Four of them required invasive mechanical ventilation. Fortunately, there was no mortality and all children had a complete recovery [12]. Another rare complication of Golytely was reported in the case of a child who developed urticaria after undergoing bowel irrigation post-ingestion of an alkali battery. It resolved with the stopping of the irrigation and administration of anti-histamine [13]. This rare complication was also reported in an adult patient [14].

One of the major concerns of using Golytely is the risk of electrolyte disturbances, especially dysnatremia. Although many studies have shown the safety of PEG regarding electrolytes balance, there have been a few reported cases describing significant dysnatremia. Ten cases of severe hyponatremia after the use of PEG were reported until 2017. All of them were adults and their ages ranged between 50-70 years. Their presentation was predominantly neurological with seizure being the most common clinical feature. One patient developed cardiac arrest and died [15]. In another case series, two adult patients developed hypernatremia. One of them had presented with massive diarrhea, seizure, and aspiration pneumonia, while the other patient had presented mainly with neurological symptoms. Unfortunately, both of them developed cardiac arrest and passed away [16]. In an adult case reported more recently, the patient developed cardiac arrest with ventricular fibrillation four hours after the consumption of Golytely. Unfortunately, due to severe neurological sequelae, the patient died 18 days later [17].

To our knowledge, this is the first reported pediatric case describing a lethal complication caused by the ingestion of mal-prepared Golytely. Our patient was administered an undiluted 375 ml of Golytely over one hour. This caused a major shift in water levels, and the child passed a very large amount of watery diarrhea afterward. As a consequence, he developed severe hypernatremic dehydration and hypovolemic shock. The hypernatremia was progressive, and the osmolarity kept worsening as well despite adequate rehydration. Later, the child developed cardiac arrest and PEA followed by severe brain edema, tonsillar herniation, and MODS. His hypernatremia and hyperosmolarity were refractory and difficult to control. Unfortunately, our patient then developed ventricular tachycardia and passed away.

## Conclusions

The appropriate and careful preparation of Golytely is a crucial step before administering it for colonic cleansing. Otherwise, it will lead to lethal complications. At our institution, after the occurrence of the incident reported in this study, the pharmacist has been assigned the responsibility to dilute Golytely in the pharmacy. After that, another two healthcare workers (nurses or doctors) are expected to double-check the preparation's appropriateness before administering the ordered volume to the patient.
